# Flavonoid and lignan intake and pancreatic cancer risk in the European prospective investigation into cancer and nutrition cohort

**DOI:** 10.1002/ijc.30190

**Published:** 2016-06-10

**Authors:** Esther Molina‐Montes, María‐José Sánchez, Raul Zamora‐Ros, H.B(as) Bueno‐de‐Mesquita, Petra A. Wark, Mireia Obon‐Santacana, Tilman Kühn, Verena Katzke, Ruth C. Travis, Weimin Ye, Malin Sund, Alessio Naccarati, Amalia Mattiello, Vittorio Krogh, Caterina Martorana, Giovanna Masala, Pilar Amiano, José‐María Huerta, Aurelio Barricarte, José‐Ramón Quirós, Elisabete Weiderpass, Lene Angell Åsli, Guri Skeie, Ulrika Ericson, Emily Sonestedt, Petra H. Peeters, Isabelle Romieu, Augustin Scalbert, Kim Overvad, Matthias Clemens, Heiner Boeing, Antonia Trichopoulou, Eleni Peppa, Pavlos Vidalis, Kay‐Tee Khaw, Nick Wareham, Anja Olsen, Anne Tjønneland, Marie‐Christine Boutroun‐Rualt, Françoise Clavel‐Chapelon, Amanda J. Cross, Yunxia Lu, Elio Riboli, Eric J. Duell

**Affiliations:** ^1^ Andalusian School of Public Health, Instituto De Investigación Biosanitaria Ibs, GRANADAHospitales Universitarios De Granada/Universidad De GranadaGranadaSpain; ^2^ CIBERESPCIBER Epidemiología Y Salud PúblicaSpain; ^3^ Section of Nutrition and MetabolismInternational Agency for Research on Cancer (IARC)LyonFrance; ^4^ National Institute for Public Health and the Environment (RIVM)BilthovenThe Netherlands; ^5^ Department of Gastroenterology and HepatologyUniversity Medical CentreUtrechtThe Netherlands; ^6^ Department of Epidemiology and Biostatistics, the School of Public HealthImperial College LondonLondonUnited Kingdom; ^7^ Department of Social and Preventive Medicine, Faculty of MedicineUniversity of MalayaKuala LumpurMalaysia; ^8^ Global eHealth Unit, Department of Primary Care and Public Health, the School of Public HealthImperial College LondonLondonUnited Kingdom; ^9^ Unit of Nutrition and Cancer, Catalan Institute of Oncology (ICO‐Idibell)BarcelonaSpain; ^10^ Division of Cancer EpidemiologyGerman Cancer Research Center (DKFZ)HeidelbergGermany; ^11^ Cancer Epidemiology Unit, Nuffield Department of Population HealthUniversity of OxfordOxfordUnited Kingdom; ^12^ Department of Medical Epidemiology and BiostatisticsKarolinska InstitutetStockholmSweden; ^13^ The Medical Biobank at Umeå UniversityUmeåSweden; ^14^ Molecular and Genetic Epidemiology UnitHuGeF—Human Genetics FoundationTorinoItaly; ^15^ Dipartimento Di Medicina Clinica E Chirurgia, Federico II UniversityNaplesItaly; ^16^ Epidemiology and Prevention Unit Fondazione IRCCS Istituto Nazionale Dei TumoriMilanItaly; ^17^ Cancer Registry ASPRagusaItaly; ^18^ Molecular and Nutritional Epidemiology UnitCancer Research and Prevention Institute—ISPOFlorenceItaly; ^19^ Public Health Division of GipuzkoaBioDonostia Research InstituteSan SebastiánSpain; ^20^ Department of EpidemiologyMurcia Regional Health Council, IMIB‐ArrixacaMurciaSpain; ^21^ Public Health Institute of NavarraPamplonaSpain; ^22^ Public Health DirectorateAsturiasSpain; ^23^ Department of Community Medicine, Faculty of Health SciencesUniversity of Tromsø, the Arctic University of NorwayTromsøNorway; ^24^ Department of ResearchCancer Registry of NorwayOsloNorway; ^25^ Genetic Epidemiology Group, Folkhälsan Research CenterHelsinkiFinland; ^26^ Department of Clinical Sciences in MalmöLund UniversityLundSweden; ^27^ Department of EpidemiologyJulius Center for Health Sciences and Primary Care, University Medical Center, UtrechtThe Netherlands; ^28^ Department of Public HealthSection for Epidemiology, Aarhus UniversityAarhusDenmark; ^29^ Department of EpidemiologyGerman Institute of Human Nutrition Potsdam‐RehbrueckeNuthetalGermany; ^30^ Hellenic Health FoundationAthensGreece; ^31^ WHO Collaborating Center for Nutrition and Health, Unit of Nutritional Epidemiology and Nutrition in Public Health, Department of Hygiene, Epidemiology, and Medical StatisticsUniversity of Athens Medical SchoolAthensGreece; ^32^ University of Cambridge School of Clinical MedicineCambridgeUnited Kingdom; ^33^ Epidemiology UnitMedical Research CouncilCambridgeUnited Kingdom; ^34^ Danish Cancer Society Research CenterCopenhagenDenmark; ^35^ Inserm, CESP Centre for Research in Epidemiology and Population HealthFrance

**Keywords:** diet, flavonoids, lignans, pancreatic cancer, cohort

## Abstract

Despite the potential cancer preventive effects of flavonoids and lignans, their ability to reduce pancreatic cancer risk has not been demonstrated in epidemiological studies. Our aim was to examine the association between dietary intakes of flavonoids and lignans and pancreatic cancer risk in the European Prospective Investigation into Cancer and Nutrition (EPIC) cohort. A total of 865 exocrine pancreatic cancer cases occurred after 11.3 years of follow‐up of 477,309 cohort members. Dietary flavonoid and lignan intake was estimated through validated dietary questionnaires and the US Department of Agriculture (USDA) and Phenol Explorer databases. Hazard ratios (HR) and 95% confidence intervals (CIs) were calculated using age, sex and center‐stratified Cox proportional hazards models, adjusted for energy intake, body mass index (BMI), smoking, alcohol and diabetes status. Our results showed that neither overall dietary intake of flavonoids nor of lignans were associated with pancreatic cancer risk (multivariable‐adjusted HR for a doubling of intake = 1.03, 95% CI: 0.95–1.11 and 1.02; 95% CI: 0.89–1.17, respectively). Statistically significant associations were also not observed by flavonoid subclasses. An inverse association between intake of flavanones and pancreatic cancer risk was apparent, without reaching statistical significance, in microscopically confirmed cases (HR for a doubling of intake = 0.96, 95% CI: 0.91–1.00). In conclusion, we did not observe an association between intake of flavonoids, flavonoid subclasses or lignans and pancreatic cancer risk in the EPIC cohort.

## Abstract 2

**What's new?**

Flavonoids and lignans found in plant‐based foods are potent cancer chemopreventive agents but little is known about their effects on pancreatic cancer risk. Here the authors address this question in a large prospective epidemiological study using comprehensively derived dietary data. Their results support growing evidence that there is no association between food‐based consumption of both substances with pancreatic cancer risk.

Pancreatic cancer incidence and mortality estimates for the year 2012 in Europe show that the prognosis of this cancer remains poor (mortality:incidence ratio is 0.98).^1^ Age, sex, family history, smoking, chronic pancreatitis, obesity and diabetes are established risk factors,^2^ but no dietary factors have been classified as convincingly associated with pancreatic cancer risk.^3^ Identification of dietary factors, including bioactive compounds naturally present in food, are increasingly the focus of investigation to reduce the burden of this cancer.^4^

Flavonoids and its subclasses (flavones, flavonols, flavanones, flavanols—including flavan‐3‐ol monomers, proanthocyanidins, and theaflavins—anthocyanidins, and isoflavones) are bioactive compounds with phenolic structures commonly present in fruits, vegetables and plant‐based beverages.^5^ These antioxidant compounds exert putative anticarcinogenic effects through a wide range of molecular mechanisms.^6^ Epidemiological evidence of their role in cancer prevention is, however, still inconsistent. For instance, some studies point to a reduced risk of various smoking‐related cancers,^7^, ^8^ while others have not demonstrated an inverse association with cancer risk.^9^, ^10^ Lignans are another diverse group of polyphenols present in foods of plant origin and one of the major classes of phytoestrogens. They also seem to have anticancer activity, possibly *via* pro‐estrogenic mechanisms or antioxidant effects,^5^ although fewer studies have examined their cancer preventive effects.^11^

The anticancer effects of flavonoids on pancreatic cancer have been widely researched in *in vitro* and *in vivo* studies. These studies suggest that flavonoids inhibit proliferation of various pancreatic cancer cell lines through induction of apoptosis and inhibition of cell growth.^4^ Epidemiological studies examining the association between flavonoids on pancreatic cancer risk include seven prospective studies,^9^, ^12^, ^13^, ^14^, ^15^, ^16^, ^17^ and one case‐control study.^18^ A flavonoid‐rich food pattern and the intake of flavonols were both associated with a reduced pancreatic cancer risk in the Multiethnic cohort study (MEC), with this association being more prominent in smokers.^17^, ^19^ This finding was, however, not confirmed in a Finnish cohort study, except when smokers not consuming antioxidant supplements were considered.^14^ The case‐control study conducted in Italy reported an inverse association with pancreatic cancer risk for proanthocyanidin intake.^18^ In contrast, findings of a large prospective study that included 2,379 pancreatic cancer cases (within the National Institutes of Health‐AARP Diet and Health Study, NIH‐AARP) did not support an association between flavonoid intake and pancreatic cancer risk.^13^ The other studies, which had smaller sample sizes, also did not observe significant associations for the intake of total flavonoids or subclasses with pancreatic cancer risk. The association between dietary intake of lignans and pancreatic cancer risk has not yet been examined in epidemiological studies.

A compelling argument of a possible lack of association between dietary flavonoids and lignans with pancreatic cancer risk is that fruits, their major food sources, seem not to play a role in the aetiology of this cancer.^20^ However, an association between intake of flavonoids and lignans with pancreatic cancer risk is plausible given their observed preventative effects against diabetes.^21^, ^22^

Our aim was to examine the association between dietary intakes of flavonoids and lignans and pancreatic cancer risk in the European Prospective Investigation into Cancer and Nutrition (EPIC) cohort, in which a diverse intake of these compounds was described.^23^, ^24^

## Methods

### Study design

The EPIC study is a multicenter prospective cohort study that is being carried out in 23 centers from 10 European countries (Denmark, France, Germany, Greece, Italy, Norway, Spain, Sweden, The Netherlands and United Kingdom). Recruitment of over half a million participants, aged 25–70 years, took place between 1992 and 2000. The majority of the participants were recruited from the general population although some centers also constituted their cohorts with local blood donors (Spain and Italy), vegetarians (the health conscious cohort of Oxford in the UK), women attending breast cancer screening programs (Florence in Italy and Utrecht in The Netherlands), or women belonging to a health insurance scheme for state school and university employees (France). Participants gave their informed consent for participating in the study. The study was approved by the Internal Review Board of the International Agency for Research on Cancer, as well as by local institutions of the participating centers. Methods of recruitment have been described in detail elsewhere.^25^, ^26^

### Study population

A total of 23,785 participants with prevalent cancer at baseline other than non‐melanoma skin cancer, 4,383 participants with missing or incomplete information on follow‐up, 6,253 participants with incomplete dietary or non‐dietary information, and 9,600 participants with a ratio for energy intake versus energy expenditure in the top or bottom 1% were excluded. The final study sample consisted of 477,309 participants (29.8% men).

### Assessment of the outcome

Record linkage with population‐based cancer registries, as well as with national mortality registries, was performed to identify incident cancer cases and to assess the vital status of the participants. Complete follow‐up data was obtained until at least December 2004 and maximally to December 2008 depending on the EPIC center. Active follow‐up was carried out in Germany (up to December 2008 for Potsdam and June 2010 for Heidelberg), Greece (up to December 2009), and France (up to December 2006) by reviewing cancer, pathology and health insurance records of each participant, and also by directly contacting their next‐of‐kin.

Incident pancreatic cancer cases were defined as adenocarcinomas of the exocrine pancreas [International Classification of Diseases for Oncology, Third Edition (ICD‐O‐3), codes C25.0–C25.3, C25.7–C25.9]. Endocrine tumors (*n* = 40), secondary tumors (*n* = 67) and tumors of uncertain, benign or metastatic behavior (*n* = 3) were all censored at the date of their diagnosis. Of all 865 exocrine pancreatic cancer cases, 608 (70.3%) were microscopically confirmed, based on histology of the primary tumor (*n* = 359), histology of the metastasis (*n* = 68), cytology (*n* = 130) or autopsy (*n* = 51). Diagnosis was based on clinical symptoms, physical examination or imaging results for the remaining cases.

### Assessment of diet and lifestyle data

Country‐specific validated dietary questionnaires (DQs) were used to inquire the participants about their habitual diet over the previous year, namely: quantitative food frequency questionnaires (FFQs) in Germany, Greece, UK, Northern Italy and The Netherlands, diet history questionnaires in Spain, France and Ragusa (Italy), and semi‐quantitative FFQs in Denmark, Naples (Italy), Norway, Umea (Sweden). In Malmö (Sweden), quantitative questionnaire with a 7‐day menu book were used.^26^ The EPIC nutrient database (ENDB) was used to estimate nutrient and total energy intake.^27^ Intake of total flavonoids and its subclasses (flavones, flavonols, flavanones, flavanols—including flavan‐3‐ol monomers, proanthocyanidins, and theaflavins—anthocyanidins, and isoflavones) as well as intake of lignans was estimated through databases on content of polyphenols in foods.^28^, ^29^, ^30^ Effects of food processing and cooking on polyphenol content were considered by reported values in the above mentioned databases, or by applying retention factors.^31^ Information on dietary flavonoids and lignans was available for 1,877 food items. More details on the estimation of the dietary intake of flavonoids and lignans have been described elsewhere.^23^, ^24^

Other lifestyle data were collected at recruitment using standardized questionnaires including self‐reported diabetes mellitus status, lifetime history of smoking and alcohol consumption, physical activity and socio‐economic status. Approximately half of all diabetes self‐reported cases were included in a validation study, which consisted of verifying this diagnosis using additional sources of information, such as use of diabetes‐related medication, repeated self‐report during follow‐up, or linkage to diabetes registries and patient records.^32^ Regarding anthropometrics, participant's height and weight were measured in all EPIC centers except in Norway, France and in a subgroup of the Oxford cohort where these data were self‐reported. Measurements also included waist circumference except in Norway and Umea and in the Oxford cohort where these data were self‐reported.^26^ This data was corrected for differences in clothing and also for self‐reports through prediction equations based on real measures in a subsample of the French and Oxford cohorts.^33^

### Statistical analysis

Cox proportional hazards regression models were used to estimate hazard ratios (HRs) and 95% confidence intervals (CIs) for intake of total flavonoids, flavonoid subclasses and lignans associated with pancreatic cancer risk. Regression models were stratified by age at recruitment in 1‐year categories, sex and center to additionally control for between‐center differences in the dietary assessment methods used at recruitment and differences in follow‐up procedures. Time at entry was age at recruitment, and time at exit was age at first pancreatic cancer diagnosis for cases and age at censoring for non‐cases (death, loss to follow‐up, or end of the follow‐up, whichever came first). The proportional hazard assumption was satisfied as was verified after including time dependent covariates in the Cox models and testing for a non‐zero slope in a linear regression of the scaled Schoenfeld residuals on functions of time.^34^ Intake of dietary flavonoids and lignans were modelled as categorical variables using cohort‐wide quintiles and considering the first quintile as the referent. The trend of association across quintiles was evaluated by using a linear variable of the quintile‐specific medians of the dietary intakes.

Intakes were also modelled on a continuous log_2_ scale to estimate risks associated with a doubling of intake and to normalize the skewed data. We previously examined the shape of the dose–response relationship by fitting Cox models with restricted cubic splines using three knots on the 5th, 50th, and 95th percentile of the distribution of flavonoids and lignan intakes.^35^ These models suggested a linear relationship for total flavonoids, flavonoid subclasses, and for lignans. Linearity was maintained with a larger number of knots.

Covariates considered *a priori* as factors associated with pancreatic cancer risk and dietary intake of flavonoids and lignans were tested for confounding by comparing models with and without each variable. Those variables that changed estimates >10% or significantly improved the model fit likelihood ratio test were retained in the regression model. Physical activity, educational level, waist circumference and dietary factors possibly associated with pancreatic cancer risk (folate, fiber, saturated fatty acids, red and processed meat) did not comply with these criteria and were therefore not retained. The associations of dietary flavonoids and lignans intakes with pancreatic cancer risk were examined in crude models (stratified by age, sex, and center), which were further adjusted (Model 1) for energy intake from fat and from non‐fat sources, and additionally (Model 2) for body mass index (BMI), smoking status, alcohol intake, and diabetes status at recruitment.

We conducted stratified analyses and tested interaction using the log‐likelihood test in regression models with and without multiplicative interaction terms to examine the modifying effect of sex, BMI (normal *vs*. overweight and/or obese, considering WHO criteria^36^), waist circumference (normal *vs*. moderate and large, considering NCEP/ATPIII criteria^37^), smoking status (never *vs*. former *vs*. current smoker), median age at diagnosis (<60 *vs*. ≥60 years), and heterogeneity by country and region (mediterranean *vs*. non‐mediterranean). The potential interaction between intake of flavonoids and lignans and smoking status was further explored on an additive scale, using a five‐category variable for flavonoids (quintiles) and a three‐category variable for smoking status (never, former and current smokers).

Sensitivity analyses were conducted by excluding the first 2 years of follow‐up (excluding 88 cases) to evaluate whether reverse causation driven by early effects of subclinical disease distorted the associations, and excluding the microscopically non‐confirmed cases (*n* = 257) to minimize possible misclassification of tumors. We also stratified the associations by diabetes status to explore whether diabetes mediates and/or moderates the possible association between dietary intake of flavonoids and lignans with risk of pancreatic cancer. However, the associations could only be examined in non‐diabetics, including 773 pancreatic cancer cases, due to insufficient number of pancreatic cancer cases with diabetes at recruitment (55 cases).

Stata statistical software was used for the data analysis (release 12.0; College Station, TX: Stata Corp LP, 2005). Statistical significance was based on two‐sided *p* values < 0.05.

## Results

In total, 865 pancreatic cancer cases (45.8% in men) were documented after 11.3 years of follow‐up. Median intake of flavonoids in men and women was 335.0 and 332.2 mg day^−1^, respectively. Intakes were highest in the UK, possibly due to the high proportion of health conscious participants and tea drinkers in this cohort, and lowest in Norwegian women and Swedish men and women. For lignans (median intakes: 1.46 mg day^−1^ in men and 1.23 mg day^−1^ in women), a similar pattern was observed. The main contributors to the intake of total flavonoids were flavanols (82.1%) followed by flavonols (6.1%) in men, and flavanols (80.8%) followed by anthocyanidins (6.8%) in women. The composition of flavonoids subclasses in the diet differed by country, with flavanols being overall the most frequently consumed flavonoid subclass (Table 1).

**Table 1:** Descriptive information of the EPIC cohort by country, sex, distribution of pancreatic cancer cases, and dietary intake of total flavonoids and lignans

			Intake: median, mg day^−1^ (IQR)		
	Cases (n)	Cohort sample (n)	Flavonoids	Lignans	Main flavonoid subclasses (% of total flavonoids)
**Men**					
Italy	27	14,029	398.8 (283.6–541.3)	1.12 (0.88–1.41)	Flavanols (77.5%), Flavanones (8.2%), Anthocyanidins (8.2%), Flavonols (5%)
Spain	28	15,148	423.7 (284.4–612.6)	1.06 (0.80–1.38)	Flavanols (75.5%), Flavanones (8.9%), Anthocyanidins (8%), Flavonols (6.2%)
UK	51	22,851	823.6 (556.7–1119.4)	1.99 (1.54–2.51)	Flavanols (89.3%), Flavonols (5%), Anthocyanidins (3%), Flavanones (1.7%)
The Netherlands	15	9,639	286.6 (175.6–438.7)	1.19 (0.96–1.46)	Flavanols (78.9%), Anthocyanidins (6.9%), Flavonols (6.8%), Flavanones (6.2%)
Greece	20	10,807	267.6 (196.5–354.2)	1.38 (1.07–1.85)	Flavanols (65.8%), Flavanones (12.5%), Anthocyanidins (9.8%), Flavonols (9%)
Germany	64	21,172	304.4 (199.1–473.2)	1.72 (1.29–2.23)	Flavanols (80.4%), Anthocyanidins (7.9%), Flavonols (5.5%), Flavanones (4.6%)
Sweden	73	22,309	197.0 (127.9–310.5)	0.97 (0.74–1.25)	Flavanols (78.7%), Anthocyanidins (8.1%), Flavonols (6.6%), Flavanones (5.2%)
Denmark	118	26,294	278.6 (168.8–599.2)	2.12 (1.69–2.67)	Flavanols (84.8%), Flavonols (7.5%), Flavanones (3.7%), Anthocyanidins (3.4%)
All	396	142,249	335.0 (200.9–596.2)	1.46 (1.04–2.06)	Flavanols (82.1%), Flavonols (6.1%), Anthocyanidins (5.7%), Flavanones (4.9%)
**Women**					
France	46	67,385	395.5 (272.9–606.1)	1.32 (0.99–1.71)	Flavanols (76.9%), Anthocyanidins (10.9%), Flavonols (7.7%), Flavanones (3.4%)
Italy	41	30,512	333.5 (241.5–449.4)	0.98 (0.77–1.25)	Flavanols (79.2%), Flavanones (8.3%), Anthocyanidins (6.6%), Flavonols (5.1%)
Spain	31	24,854	286.6 (191.8–410.3)	0.83 (0.61–1.13)	Flavanols (73.5%), Flavanones (12.4%), Anthocyanidins (6.6%), Flavonols (6.2%)
UK	72	52,543	707.5 (448.1–1024.8)	2.07 (1.60–2.62)	Flavanols (87.3%), Flavonols (5.3%), Anthocyanidins (3.7%), Flavanones (2.5%)
The Netherlands	52	26,866	414.5 (259.2–603.4)	1.08 (0.89–1.30)	Flavanols (80.2%), Anthocyanidins (6.6%), Flavanones (6.3%), Flavonols (5.8%)
Greece	16	15,225	231.7 (167.2–308.7)	1.17 (0.89–1.59)	Flavanols (66.3%), Flavanones (13.1%), Anthocyanidins (9.2%), Flavonols (8.5%)
Germany	41	27,411	327.1 (214.6–497.0)	1.32 (1.03–1.68)	Flavanols (80.1%), Anthocyanidins (8.2%), Flavonols (4.9%), Flavanones (4.8%)
Sweden	79	26,374	217.0 (145.4–326.8)	0.90 (0.69–1.17)	Flavanols (77.9%), Anthocyanidins (7.7%), Flavanones (6.7%), Flavonols (6.1%)
Denmark	71	28,722	387.3 (210.6–735.6)	1.80 (1.43–2.29)	Flavanols (86.4%), Flavonols (6.5%), Flavanones (3.8%), Anthocyanidins (2.8%)
Norway	20	35,168	137.5 (92.1–196.2)	0.92 (0.71–1.20)	Flavanols (70.5%), Anthocyanidins (11.7%), Flavonols (8.6%), Flavanones (8.5%)
All	469	335,060	332.2 (201.2–581.3)	1.23 (0.89–1.73)	Flavanols (80.8%), Anthocyanidins (6.8%), Flavonols (6.3%), Flavanones (5%)

The cohorts from Norway, France, Naples (Italy) and Utrecht (the Netherlands) were comprised of women only.

Data on total intake by subclasses and main food sources, using the 24‐hr recall data, can be found in Ref. 23 and 24.

Main food sources were: Total flavonoids: fruits (40%), Flavanols: tea (44%), Flavan‐3‐ols: tea (84%), Proanthocyanidins: fruits (53%), theaflavins: tea (100%), Anthocyanidins: fruits (52%), Flavonols: tea (26%), Flavanones: fruits, (50%), Flavones: tea (30%), Isoflavones: soya products (40%), lignans: vegetables (24%).

EPIC: European Prospective Investigation into Cancer and Nutrition study.

IQR: interquartile range.

European Commission (DG‐SANCO); International Agency for Research on Cancer; Danish Cancer Society (Denmark); Ligue Contre le Cancer; Institut Gustave Roussy; Mutuelle Générale de l'Education Nationale; Institut National de la Santé et de la Recherche Médicale (INSERM) (France); German Cancer Aid; German Cancer Research Center (DKFZ); Federal Ministry of Education and Research (BMBF); Deutsche Krebshilfe; Deutsches Krebsforschungszentrum; Federal Ministry of Education and Research (Germany); the Hellenic Health Foundation (Greece); Associazione Italiana per la Ricerca sul Cancro‐AIRC‐Italy; National Research Council (Italy); Dutch Ministry of Public Health, Welfare and Sports (VWS); Netherlands Cancer Registry (NKR); LK Research Funds; Dutch Prevention Funds; Dutch ZON (Zorg Onderzoek Nederland); World Cancer Research Fund (WCRF); Nordforsk; Nordic Centre of Excellence Programme on Food, Nutrition and Health (Norway); Swedish Cancer Society; Swedish Research Council and County Councils of Skåne and Västerbotten (Sweden)

The characteristics of the study population are described in Table 2. Briefly, participants in the fifth quintile of dietary intake of flavonoids and lignans were older, had a lower BMI and waist circumference, and were less likely to be smokers and diabetics compared to participants in the first quintile. Participants with higher dietary intakes of flavonoids and lignans also consumed higher amounts of alcohol, achieved a higher educational level and were more physically active. All dietary factors increased across quintiles of intake of total flavonoids and lignans, except intake of red and processed meat.

**Table 2:** Demographic and lifestyle characteristics by quintiles of dietary intake of total flavonoids and lignans

Characteristics	Quintiles of total flavonoids intake (mg day^−1^)					Quintiles of lignan intake (mg day^−1^)				
	Q1: < 176.3	Q2: 176.3–276.4	Q3: 276.4–405.5	Q4: 405.5–659.0	Q5: > 659.0	Q1: < 0.9	Q2: 0.9–1.1	Q3: 1.1–1.5	Q4: 1.5–2.0	Q5: > 2.0
**Total median intake,** mg day^−1^ (IQR)	124.2 (91.1–151.0)	225.9 (201.1–250.8)	333.0 (303.6–366.7)	515.1 (453.1–585.5)	933.9 (752.7–1138.3)	0.69 (0.57–0.78)	1.00 (0.93–1.07)	1.29 (1.21–1.38)	1.70 (1.58–1.84)	2.48 (2.20–2.94)
**Age**, years (mean±SD)	50.8 ± 9.1	51.2 ± 9.6	50.9 ± 9.6	51.3 ± 9.9	51.8 ± 11.3	51.1 ± 9.5	50.8 ± 9.5	51.0 ± 9.7	51.5± 9.9	51.7 ± 10.9
**Education > secondary school**, *n* (%)	31,797 (33.3)	39,234 (41.1)	44,937 (47.1)	47,058 (49.3)	47,959 (50.2)	32,533 (34.1)	41,423 (43.3)	44,994 (47.1)	45,929 (48.1)	46,106 (48.3)
**BMI**, kg m^−2^ (mean ± SD)	25.7 ± 4.4	25.8 ± 4.4	25.6 ± 4.3	25.3 ± 4.2	24.8 ± 4.0	25.8 ± 4.5	25.4 ± 4.3	25.3 ± 4.2	25.2 ± 4.2	25.3 ± 4.1
**Waist circumference**, cm (mean± SD)	87.5 ± 13.2	86.4 ± 13.1	85.6 ± 12.9	84.7 ± 12.8	82.8 ± 12.6	85.7 ± 2.8	85.3 ± 12.8	85.3 ± 12.8	85.1 ± 13.1	84.9 ± 13.4
**Physical activity > Moderate**, *n* (%)	27,481 (28.8)	34,911 (36.6)	38,546 (40.4)	42,213 (44.2)	44,001 (46.1)	26,564 (27.8)	34,146 (35.7)	38,757 (40.6)	41,800 (43.8)	45,885 (48.1)
**Current smoker**, *n* (%)	32,469 (34.0)	23,354 (24.5)	19,725 (20.7)	17,092 (17.9)	14,448 (15.1)	24,939 (26.1)	22,298 (23.4)	20,935 (21.9)	19,676 (20.6)	19,237 (20.1)
**Alcohol intake**, median g (IQR)	2.4 (0.4–8.8)	4.6 (0.7–13.1)	6.0 (1.0–16.3)	7.4 (1.5–20.1)	7.2 (1.6–17.6)	1.6 (0–7.0)	3.8 (0.6–12.2)	5.7 (1.2–15.3)	7.6 (1.9–18.2)	9.8 (2.5–23.8)
**Dietary intake,** (mean ± SD)										
Total energy intake (kcal)	1790.8 ± 545.1	2009.7 ± 569.3	2141.5 ± 597.7	2211.1 ± 638.1	2216.9 ± 635.1	1735.7 ± 514.8	1973.9 ± 544.3	2105.5 ± 585.2	2193.9 ± 610.8	2361.1 ± 646.7
Carbohydrates (g)	192.8 ± 63.2	217.6 ± 67.5	234.0 ± 71.3	244.1 ± 75.9	251.3 ± 78.0	191.1 ± 62.7	217.4 ± 67.1	231.0 ± 71.5	240.3 ± 73.9	260.1 ± 77.3
Fat (g)	72.8 ± 27.6	80.8 ± 28.6	84.7 ± 30.0	85.3 ± 30.9	83.1 ± 30.3	69.9 ± 25.5	78.3 ± 26.6	83.1 ± 28.8	85.8 ± 30.7	89.5 ± 33.2
Saturated fat (g)	28.5 ± 12.4	30.8 ± 12.6	32.0 ± 12.9	32.3 ± 13.1	32.2 ± 13.3	27.0 ± 11.8	30.0 ± 11.9	31.8 ± 12.5	33.0 ± 13.1	33.9 ± 14.1
Vitamin C (mg)	79.6 ± 39.5	116.4 ± 52.2	137.4 ± 64.0	149.4 ± 74.9	153.0 ± 80.4	81.7 ± 39.7	111.6 ± 47.5	130.3 ± 57.0	142.5 ± 68.0	169.6 ± 89.5
Vitamin E (mg)	9.6 ± 4.7	11.7 ± 5.3	12.8 ± 5.7	13.3 ± 5.9	13.4 ± 6.2	9.4 ± 4.36	11.3 ± 4.8	12.5 ± 5.4	13.1 ± 5.8	14.5 ± 6.7
Dietary folate (µg)	227.0 ± 75.1	277.1 ± 86.9	308.5 ± 100.8	335.8 ± 113.7	392.9 ± 141.4	213.0 ± 67.1	264.7 ± 73.2	301.3 ± 82.5	338.9 ± 96.7	423.5 ± 143.3
Dietary fiber (g)	18.1 ± 5.9	21.1 ± 6.2	23.2 ± 6.6	24.9 ± 7.6	26.5 ± 9.0	16.9 ± 5.1	20.6 ± 5.4	22.8 ± 6.2	24.7 ± 6.9	28.9 ± 8.7
Red and processed meat (g)	96.8 ± 55.6	101.6 ± 57.3	102.6 ± 58.7	102.2 ± 62.4	89.5 ± 65.4	88.9 ± 50.7	95.6 ± 52.1	100.2 ± 55.8	103.3 ± 61.5	104.5 ± 76.1
Red meat (g)	40.2 ± 34.2	44.5 ± 35.2	44.6 ± 35.3	45.4 ± 36.5	40.7 ± 37.6	34.7 ± 29.7	40.6 ± 31.6	44.0 ± 33.4	46.8 ± 36.3	49.3 ± 44.4
Fruits and vegetables (g)	250.5 ± 147.2	394.3 ± 198.3	480.2 ± 239.8	532.6 ± 283.3	561.1 ± 324.6	281.1 ± 156.6	385.0 ± 191.6	449.6 ± 228.3	493.1 ± 265.5	609.9 ± 350.9
**Diabetes status**, *n* (%) Yes, diagnosis verified	1,405 (1.5)	1,381 (1.4)	1,237 (1.3)	1,153 (1.2)	707 (0.7)	1,088 (1.1)	1,143 (1.2)	1,090 (1.1)	1,222 (1.3)	1,340 (1.4)
Yes, diagnosis self‐reported	1,665 (1.7)	1,626 (1.7)	1,479 (1.5)	1,336 (1.4)	1,324 (1.4)	1,996 (2.1)	1,562 (1.6)	1,382 (1.4)	1,273 (1.3)	1,217 (1.3)

All dietary intakes, including total flavonoids, are estimated from the FFQ and not energy‐adjusted.

Missing data on Educational level for 1,260 men and 4,964 women; Physical activity for 3,075 men and 38,956 women; Waist circumference for 12,043 men and 94,998 women; Smoking status for 1,959 men and 7,774 women; Diabetes status at recruitment for 3,096 men and 13,745 women.

Neither dietary intake of total flavonoids nor of lignans were associated with risk of pancreatic cancer (HR for a doubling of intake after a log_2_ transformation = 1.03, 95% CI: 0.95–1.11 and 1.02; 95% CI: 0.89–1.17, respectively) (Table 3). None of the subclasses of flavonoids showed a statistically significant association with pancreatic cancer risk, although results of flavonols, flavan‐3‐ol monomers and theaflavins suggested a positive association across quantiles (*p* trend = 0.06, 0.05, and 0.04, respectively). Risk of pancreatic cancer risk tended to decrease with higher intake of flavanones (HR Q5 *vs*. Q1 = 0.78, 95% CI: 0.61–0.99, *p* trend = 0.07), but not so after multivariable adjustment (*p* trend = 0.24). The associations were all not statistically significant on the continuous scale. Adjustment for smoking status affected the associations most, while adjustment by the other variables had barely an effect.

**Table 3:** Hazard ratios (95% confidence intervals) of pancreatic cancer by cohort wide quintiles of dietary intake of total flavonoids intake and its subclasses, and lignans

	Quintile of intake						Intake as continuous variable (log2)
	Q1	Q2	Q3	Q4	Q5	*p* for trend	
**Total Flavonoids (mg day** ^−1^ **)**	<176.3	176.3–276.4	276.4–405.5	405.5–659.0	>659.0		
Median intake (mg day^−1^)	124.2	225.9	333.0	515.1	933.9		
Cases/PY	182/1,044,264	172/1,050,780	157/1,057,203	169/1,055,485	185/1,055,266		
HR (95% CI) Crude	1.00	0.93 (0.75–1.15)	0.91 (0.73–1.14)	0.96 (0.77–1.21)	0.99 (0.78–1.26)	0.783	0.99 (0.92–1.07)
HR (95% CI) Model 1	1.00	0.92 (0.74–1.14)	0.89 (0.70–1.11)	0.93 (0.73–1.18)	0.95 (0.74–1.22)	0.999	0.98 (0.91–1.06)
HR (95% CI) Model 2	1.00	0.98 (0.79–1.22)	0.98 (0.78–1.23)	1.06 (0.84–1.35)	1.10 (0.85–1.42)	0.318	1.03 (0.95–1.11)
**Flavanols (mg day** ^−1^ **)**	<122.3	122.3‐200.2	200.2–311.2	311.2–555.9	>555.9		
Median intake (mg day^−1^)	83.5	160.5	248.9	406.0	823.6		
Cases/person‐years	171/1,041,548	174/1,050,550	160/1,056,609	175/1,058,220	185/1,056,072		
HR (95% CI) Crude	1.00	1.00 (0.81–1.24)	0.96 (0.76–1.20)	1.03 (0.82–1.30)	1.01 (0.79–1.29)	0.834	0.98 (0.92–1.04)
HR (95% CI) Model 1	1.00	0.99 (0.79–1.22)	0.94 (0.74–1.18)	1.01 (0.80–1.27)	0.98 (0.76–1.26)	0.977	0.99 (0.93–1.05)
HR (95% CI) Model 2	1.00	1.05 (0.84–1.30)	1.03 (0.82–1.30)	1.14 (0.90–1.45)	1.13 (0.87–1.46)	0.339	0.97 (0.91–1.04)
**Flavan‐3‐ols (mg day** ^−1^ **)**	<19.3	19.3–33.8	33.8–79.8	79.8–376.0	>376.0		
Median intake (mg day^−1^)	13.0	25.7	46.4	170.3	525.1		
Cases/PY	152/1,029,952	172/1,056,221	171/1,055,170	163/1,063,602	207/1,058,053		
HR (95% CI) Crude	1.00	0.98 (0.78–1.23)	0.93 (0.74–1.18)	0.95 (0.74–1.21)	1.10 (0.84–1.43)	0.244	1.00 (0.96–1.05)
HR (95% CI) Model 1	1.00	0.96 (0.77–1.21)	0.90 (0.70–1.14)	0.91 (0.71–1.18)	1.06 (0.81–1.39)	0.285	1.00 (0.96–1.05)
HR (95% CI) Model 2	1.00	1.03 (0.82–1.29)	0.96 (0.75–1.22)	1.01 (0.77–1.30)	1.23 (0.93–1.62)	0.052	1.02 (0.98–1.07)
**Proanthocyanidins (mg day** ^−1^ **)**	<82.8	82.8–125.9	125.9–171.9	171.9–239.4	>239.4		
Median intake (mg day^−1^)	57.6	104.2	148.3	200.2	312.3		
Cases/PY	203/1,057,709	182/1,058,642	159/1,048,258	147/1,045,176	174/1,053,213		
HR (95% CI) Crude	1.00	0.89 (0.73–1.09)	0.79 (0.64–0.98)	0.80 (0.63–1.00)	0.96 (0.77–1.21)	0.823	0.95 (0.88–1.01)
HR (95% CI) Model 1	1.00	0.88 (0.71–1.07)	0.77 (0.62–0.96)	0.77 (0.61–0.97)	0.90 (0.71–1.16)	0.524	0.92 (0.86–1.01)
HR (95% CI) Model 2	1.00	0.94 (0.76–1.15)	0.85 (0.68–1.05)	0.86 (0.68–1.09)	1.02 (0.80–1.31)	0.777	0.96 (0.89–1.04)
**Theaflavins (mg day** ^−1^ **)**	<0.01	0.01–1.67	1.6–13. 6	>13. 6			
Median intake (mg day^−1^)	0	0.4	5.9	19.3			
Cases/PY	325/2,121,372	175/1,031,030	158/1,052,172	207/1,057,739			
HR (95% CI) Crude	1.00	1.08 (0.85–1.36)	1.02 (0.80–1.30)	1.19 (0.91–1.54)		0.236	1.00 (0.99–1.02)
HR (95% CI) Model 1	1.00	1.07 (0.85–1.36)	1.02 (0.79–1.30)	1.18 (0.91–1.53)		0.261	1.00 (0.98–1.02)
HR (95% CI) Model 2	1.00	1.13 (0.89–1.43)	1.11 (0.87–1.42)	1.35 (1.03–1.75)		0.042	1.01 (0.99–1.02)
**Anthocyanidins (mg day** ^−1^ **)**	<11.4	11.4–18.6	18.6–27.2	27.2–41.6	>41.6		
Median intake (mg day^−1^)	7.8	15.0	22.5	33.1	56.1		
Cases/PY	215/1,068,092	188/1,064,395	183/1,053,542	148/1,044,414	131/1,032,556		
HR (95% CI) Crude	1.00	0.95 (0.78–1.16)	1.02 (0.83–1.25)	0.94 (0.76–1.18)	0.98 (0.77–1.26)	0.904	0.97 (0.91–1.03)
HR (95% CI) Model 1	1.00	0.94 (0.77–1.15)	0.99 (0.81–1.22)	0.91 (0.72–1.15)	0.93 (0.72–1.22)	0.625	0.95 (0.89–1.02)
HR (95% CI) Model 2	1.00	1.01 (0.82–1.23)	1.08 (0.88–1.33)	1.00 (0.79–1.27)	1.02 (0.78–1.33)	0.967	0.99 (0.92–1.06)
**Flavonols (mg day** ^−1^ **)**	<13.2	13.2–19.0	19.0–26.6	26.6–39.3	>39.3		
Median intake (mg day^−1^)	10.1	16.0	22.4	32.3	52.1		
Cases/PY	165/1,056,465	179/1,057,888	174/1,054,865	157/1,047,858	190/1,045,922		
HR (95% CI) Crude	1.00	1.05 (0.85–1.30)	1.06 (0.85–1.33)	1.03 (0.80–1.31)	1.22 (0.95–1.57)	0.120	1.00 (0.91–1.10)
HR (95% CI) Model 1	1.00	1.04 (0.83–1.29)	1.04 (0.82–1.31)	1.00 (0.78–1.29)	1.19 (0.91–1.55)	0.199	0.98 (0.89–1.08)
HR (95% CI) Model 2	1.00	1.10 (0.88–1.37)	1.11 (0.88–1.41)	1.08 (0.84–1.40)	1.31 (1.00–1.72)	0.057	1.02 (0.93–1.13)
**Flavanones (mg day** ^−1^ **)**	<5.7	5.7–11.9	11.9–20.0	20.0–33.0	>33.0		
Median intake (mg day^−1^)	3.2	8.5	15.7	25.7	46.3		
Cases/PY	194/1,053,391	168/1,039,283	168/1,042,132	186/1,062,831	149/1,065,361		
HR (95% CI) Crude	1.00	0.93 (0.75–1.15)	0.97 (0.78–1.20)	1.00 (0.82–1.24)	0.81 (0.64–1.02)	0.127	0.97 (0.93–1.01)
HR (95% CI) Model 1	1.00	0.92 (0.74–1.14)	0.95 (0.77–1.18)	0.98 (0.79–1.21)	0.78 (0.61–0.99)	0.072	0.96 (0.92–1.00)
HR (95% CI) Model 2	1.00	0.96 (0.78–1.19)	1.01 (0.81–1.25)	1.05 (0.85–1.30)	0.84 (0.66–1.07)	0.235	0.98 (0.94–1.02)
**Flavones (mg day** ^−1^ **)**	<1.1	1.1–2.0	2.0–3.0	3.0–5.0	>5.0		
Median intake (mg day^−1^)	0.7	1.5	2.5	3.8	7.3		
Cases/PY	184/1,069,119	180/1,055,741	179/1,053,937	171/1,046,300	151/1,037,902		
HR (95% CI) Crude	1.00	0.99 (0.79–1.23)	1.01 (0.80–1.27)	1.05 (0.83–1.32)	0.88 (0.68–1.14)	0.324	0.98 (0.93–1.04)
HR (95% CI) Model 1	1.00	0.98 (0.78–1.22)	0.99 (0.79–1.25)	1.02 (0.81–1.30)	0.85 (0.66–1.11)	0.227	0.98 (0.92–1.03)
HR (95% CI) Model 2	1.00	1.06 (0.85–1.32)	1.10 (0.87–1.39)	1.15 (0.90–1.47)	0.95 (0.73–1.25)	0.567	1.00 (0.95–1.07)
**Isoflavones (mg day** ^−1^ **)**	<0.3	0.3–0.4	0.4–0.7	0.7–1.4	>1.4		
Median intake (mg day^−1^)	0.2	0.3	0.5	0.9	2.6		
Cases/PY	143/996,022	187/1,063,453	210/1,072,943	179/1,073,325	146/1,057,255		
HR (95% CI) Crude	1.00	0.88 (0.69–1.12)	0.98 (0.76–1.26)	0.88 (0.66–1.16)	0.98 (0.72–1.35)	0.639	0.99 (0.92–1.07)
HR (95% CI) Model 1	1.00	0.86 (0.67–1.10)	0.94 (0.72–1.21)	0.82 (0.61–1.11)	0.91 (0.64–1.28)	0.903	0.97 (0.90–1.05)
HR (95% CI) Model 2	1.00	0.84 (0.66–1.08)	0.90 (0.69–1.17)	0.81 (0.61–1.09)	0.91 (0.64–1.28)	0.750	0.97 (0.90–1.05)
**Lignans (mg day** ^−1^ **)**	<0.9	0.9–1.1	1.1–1.5	1.5–2.0	>2.0		
Median intake (mg day^−1^)	0.7	1.0	1.3	1.7	2.5		
Cases/PY	167/1,070,077	141/1,066,378	179/1,053,965	167/1,039,629	211/1,032,949		
HR (95% CI) Crude	1.00	0.86 (0.69–1.09)	1.08 (0.86–1.35)	0.90 (0.70–1.16)	1.01 (0.78–1.32)	0.728	1.03 (0.91–1.17)
HR (95% CI) Model 1	1.00	0.85 (0.67–1.07)	1.04 (0.82–1.32)	0.86 (0.66–1.12)	0.95 (0.70–1.27)	0.903	1.00 (0.88–1.15)
HR (95% CI) Model 2	1.00	0.88 (0.70–1.11)	1.09 (0.86–1.39)	0.91 (0.69–1.19)	0.99 (0.74–1.34)	0.919	1.02 (0.89–1.17)

*HR Crude*—stratified by age (1‐year categories), sex, and centre.

*HR for Model 1*—additionally adjusted for total energy intake (kcal day^−1^) from fat and from non‐fat sources (continuous).

*HR for Model 2*—additionally adjusted for body mass index in kg m^−2^ (continuous), smoking status and intensity (never; current, 1–15 cigarettes per day, 16–25 cigarettes per day, 26+ cigarettes per day; former, quit ≤10 years, quit 11–20 years, quit 20+ years; current, pipe/cigar/occasional; current/former, missing; unknown), alcohol intake (non drinkers; drinkers of 0–6 g day^−1^, >6–12 g day^−1^, >12–24 g day^−1^, >24–60 g day^−1^; women drinkers of: >60 g day^−1^; men drinkers of: >60–96 g day^−1^, >96 g day^−1^), diabetes status at recruitment (yes, diagnosis verified; yes, diagnosis self‐reported; not diabetic, missing status).

*Theaflavins were assessed in four groups since there was a large group of non‐consumers, which resulted in an unbalanced division of the aflavins in quintiles.

Group 1: 193,832; Group 2: 93,561; Group 3: 94,386; Group 4: 95,423.

PY: person‐years.

No statistically significant heterogeneity between countries was observed for the associations of total flavonoids (*p* = 0.21) and lignans (*p* = 0.81) with pancreatic cancer risk; the same applied for the analyses by regions. Interactions for total flavonoids and lignan intake were also not observed for sex (*p* = 0.64 and *p* = 0.39), BMI (*p* = 0.67 and p = 0.26), smoking status (*p* = 0.14 and *p* = 0.15), waist circumference (*p* = 0.83 and *p* = 0.66) and age (*p* = 0.47 and *p* = 0.89). There was also no evidence for interaction by these variables for any of the flavonoids subclasses (data not shown). An inverse association between intake of flavanone and pancreatic cancer risk was apparent, though not statistically significant, in smokers (HR log_2_ = 0.96, 95% CI: 0.89–1.02). Additionally controlling for smoking duration or intensity did not affect the risk estimates (Supporting Information Table 1). The combined effect of flavonoids and lignans with smoking on the risk of pancreatic cancer did not appreciably alter the associations with increasing intakes among never or former smokers with respect to smokers with the lowest category of intake (data not shown).

In the sensitivity analyses (Table 4), the associations between dietary intake of total flavonoids and lignans with pancreatic cancer risk remained almost unchanged. By flavonoids subclasses (Supporting Information Table 2), some associations changed to some extent after restricting the analyses to microscopically confirmed pancreatic cancer cases; for instance, in multivariable adjusted models, participants in the highest quintile of flavanone intake (compared to the lowest quintile) had a 29% lower pancreatic cancer risk (95% CI: 0.53–0.95, *p* trend = 0.045), although statistical significance was not reached on the continuous scale (HR log_2_: 0.96, 95% CI: 0.91–1.00). On the other hand, the positive trends observed for flavonols, flavan‐3‐ol monomers and theaflavins were all attenuated (*p* trend = 0.17, 0.18 and 0.2, respectively). The associations in nondiabetics weakened overall. Exclusion of cases diagnosed in the first 2 years of follow‐up had a minor impact on risk estimates.

**Table 4:** Sensitivity analyses on the association between dietary intakes of total flavonoids and lignans and pancreatic cancer risk

	Quintile of intake						Intake as continuous variable (log2)
	Q1	Q2	Q3	Q4	Q5	*p* for trend	
**Total Flavonoids**							
*Non‐diabetics*							
Cases	168	147	138	155	165		
HR (95% CI)	1.00	0.90 (0.71–1.13)	0.88 (0.69–1.13)	0.98 (0.77–1.26)	0.99 (0.76–1.29)	0.664	1.00 (0.92–1.08)
*Microscopically confirmed*							
Cases	155	137	113	106	97		
HR (95% CI)	1.00	1.01 (0.80–1.29)	0.99 (0.76–1.28)	1.03 (0.78–1.36)	1.15 (0.85–1.55)	0.321	1.02 (0.93–1.11)
*> 2 years follow‐up*							
Cases	160	155	141	153	168		
HR (95% CI)	1.00	1.01 (0.80–1.27)	1.00 (0.78–1.28)	1.09 (0.84–1.40)	1.12 (0.85–1.46)	0.343	1.03 (0.95–1.12)
**Lignans**							
*Non‐Diabetics*							
Cases	151	127	160	149	186		
HR (95% CI)	1.00	0.85 (0.67–1.09)	1.04 (0.81–1.34)	0.86 (0.65–1.14)	0.93 (0.68–1.28)	0.830	0.99 (0.86–1.15)
*Microscopically confirmed*							
Cases	138	104	120	110	136		
HR (95% CI)	1.00	0.84 (0.64–1.09)	1.00 (0.76–1.32)	0.86 (0.63–1.18)	1.00 (0.70–1.42)	0.798	1.03 (0.87–1.21)
*> 2 years follow‐up*							
Cases	151	126	159	146	195		
HR (95% CI)	1.00	0.86 (0.67–1.10)	1.07 (0.83–1.37)	0.88 (0.66–1.16)	1.02 (0.74–1.39)	0.722	1.02 (0.88–1.18)

Multivariable HR—adjusted for total energy intake from fat and from non‐fat sources (continuous), body mass index in kg m^−2^ (continuous), smoking status and intensity (never; current, 1–15 cigarettes per day, 16–25 cigarettes per day, 26+ cigarettes per day; former, quit ≤10 years, quit 11–20 years, quit 20+ years; current, pipe/cigar/occasional; current/former, missing; unknown), alcohol intake (non drinkers; drinkers of 0–6 g day^−1^, >6–12 g day^−1^, >12–24 g day^−1^, >24–60 g day^−1^; women drinkers of: >60 g day^−1^; men drinkers of: >60–96 g day^−1^, >96 g day^−1^), diabetes status at recruitment (yes, diagnosis verified; yes, diagnosis self‐reported; not diabetic, missing status), and stratified by age (1‐year categories), sex, and centre.

PY: person‐years.

## Discussion

The results of the present study do not support an association between dietary intakes of total flavonoids, flavonoids subclasses or lignans with risk of pancreatic cancer. Intake of flavanones tended to be inversely associated with pancreatic cancer risk only when microscopically confirmed cases were considered, but the results were not statistically significant. No other variables exerted a differential effect on these associations.

The beneficial effect of fruits and vegetables against cancer has been attributed to fiber, vitamins and to flavonoids because of their well‐established antioxidant, antimutagenic, and antiproliferative properties.^6^ With regard to pancreatic cancer, *in vitro* and *in vivo* animal studies have implicated the anticarcinogenic activity of flavonoids *via* various biological mechanisms influencing cell signalling, cell‐cycle regulation and angiogenesis, such as down regulation of NF‐kB activity with suppression of Akt activation, induction of apoptosis on BxPC‐3 in MIA PaCa‐2 and PANC‐1 cells, or inhibition of glucose uptake through down regulation of GLUT‐1 in CD18 cells.^4^, ^6^ The possible chemopreventive effects of flavonoids are, however, less evident in epidemiological studies (Supporting Information Table 3): Intake of flavonols was reported to be inversely associated with pancreatic cancer risk in the MEC study (HR comparing extreme quintiles: 0.77, 95% CI: 0.58–1.03), which included 529 pancreatic cancer cases.^17^ This trend of decreasing risk was manifest in all subclasses (quercetin, myricetin and kaempferol), with kaempferol showing the largest risk reduction (*p* trend = 0.02), and with a stronger association observed in smokers (HR = 0.41, 95% CI: 0.22–0.74). A dietary pattern regarded as rich in flavonols was also found to decrease pancreatic cancer risk in smokers participating in the MEC study, but this was not confirmed in a validation study conducted within the EPIC cohort.^19^ Increasing intake of flavonoids also decreased pancreatic cancer risk in male smokers not consuming supplemental vitamins in the alpha‐tocopherol, beta‐carotene cancer prevention (ATBC) study (*n* = 306 cases).^14^ Both were the only studies supporting an inverse association in smokers, which could be ascribed to the known anti‐oxidative effect (inhibition of CYP450 enzymes) elicited by flavonoids.^4^ We did not observe an inverse association in smokers, except a suggestive, but not statistically significant, inverse association between flavanones and pancreatic cancer risk.

Other prospective studies did not find any association between intake of flavonoids and risk of pancreatic cancer. For instance, the largest prospective study conducted so far, which included 2,379 pancreatic cancer cases of the NIH‐AARP cohort, concluded that intake of flavonoids was not associated with pancreatic cancer risk (HR comparing extreme quintiles: 1.09, 95% CI: 0.96–1.24).^13^ Two earlier studies conducted within the Iowa Women's Health Study also reported a null association for total flavonoids and seven subclasses (*n* = 230 cases),^15^ and for catechins (*n* = 130 cases).^12^ There were also no associations with pancreatic cancer either for total flavonoids or subclasses in the Women's health study (*n* = not reported),^9^ and in a Finnish population‐based survey (*n* = 29 cases),^16^ although both studies were more prone to chance findings due their smaller sample size. Finally, a case‐control study carried out in Italy, which included 326 pancreatic cancer cases, suggested an inverse association with pancreatic cancer risk for proanthocyanidins (OR comparing extreme quintiles = 041; 95% CI: 0.24–0.69, *p* trend = 0.001).^18^

Comparison between these studies is, to some extent, limited by several issues in relation to (Supporting Information Table 3): differences in the study populations, varying ranges of intake and in dietary assessment methods used, *e.g*. databases on flavonoids content in food, the number of food items used for the evaluation of intake of flavonoids, and whether the influence of food processing on flavonoids content was taken into consideration.^12^, ^13^, ^15^ In addition, in some studies, fewer flavonoids subclasses were considered to estimate intake of total flavonoids,^9^, ^14^, ^16^ while other studies focused on a single subclass,^12^, ^17^ and only few studies considered individual subclasses of flavonoids.^14^, ^15^, ^18^ Only the NIH‐AARP overcame most of these limitations by considering all subclasses of flavonoids (except proanthocyanidins), and accounting for flavonoids values of processed foods and recipes.^13^ Likewise, our study presents results obtained using both a relatively large sample size and a comprehensive estimation of intake of flavonoids. Despite the heterogeneity between studies, our results agree with the majority of the studies indicating a lack of association between dietary intake of flavonoids and risk of pancreatic cancer. The fact that *in vivo* and *in vitro* effects of flavonoids disagree with data from epidemiological studies might be due to the poor bioavailability of these compounds, to dietary intakes at levels that are under the threshold of their biological activity,^5^ or due to measurement errors in the dietary assessment methods. In fact, only two studies considered proanthocyanidins for the quantification of flavanols.^15^, ^18^ More reliable estimates of exposure through the use of biomarkers may allow a better understanding of the role of polyphenols in the aetiology of diseases, such as pancreatic cancer.^38^

A large proportion of the pancreatic cancer cases in our cohort were microscopically confirmed. An inverse association of borderline significance became apparent between flavanones and pancreatic cancer risk after restricting the analysis to these cases. The positive trend we observed for flavan‐3‐ol monomers, flavonols and theaflavins, was not consistent in all our analyses nor statistically significant, and is also not supported by the fact that the major food sources of these subclasses (tea, coffee, fruits and vegetables) are not linked to pancreatic cancer risk.^20^, ^39^, ^40^

Stratified analyses by diabetes status revealed that flavonoids and lignans were not inversely associated with pancreatic cancer in nondiabetics. In a previous study within the EPIC‐InterAct study, an inverse association between these subclasses and risk of type 2 diabetes was observed.^21^ Although dietary intake of lignans was not associated with diabetes in the EPIC‐InterAct study,^21^ gut microbiota metabolites of lignans have been associated with a lower risk of diabetes in the Nurses’ Health Study.^41^

Flavonoids and lignans may reduce the long‐term damaging effects of diabetes by improving insulin secretion, increasing glucose uptake and reducing insulin resistance,^42^ which could in turn reduce inflammation and oxidative stress induced by hyperglycaemia, and consequently reduce pancreatic cancer risk.^43^ We could not test this hypothesis due to limited number of participants with diabetes at recruitment who developed pancreatic cancer thereafter (*n* = 55).

Several strengths of this study merit consideration. The large sample size and long follow‐up enabled stratified analysis by smoking status and other potential effect modifiers with sufficient statistical power. Another major strength and distinguishing feature of our study is that we were able to minimize bias due to misclassification of cases after restricting the study population to microscopically confirmed pancreatic cancer cases. The influence of prediagnostic disease on the associations is unlikely since exclusion of cases diagnosed within the first 2 years of follow‐up did not alter the associations. Our study also benefits from an exhaustive quantification of intake of flavonoids, at a combined and individual basis for seven subclasses.^23^ This study also presents several limitations. Dietary measurement error may have led to some misclassification of the dietary exposure. The regression calibration approach to correct measurement error was, however, not suitable given that flavonoids and lignans are contained in specific foods only.^44^ It is also possible that intake of flavonoids and lignans is underestimated due to incomplete information on their content in some foods, such as thearubigins for tea, or unknown compositional data of some lignans,^23^ and because we could not consider dietary supplements (herbs/plants or their constituents) as another source of intake. The latter should not have affected our results as these types of products represented 8–17% of the supplements used in the EPIC study.^45^ Residual confounding might be present as we did not control for all potential confounding factors such as family history of pancreatic cancer, history of pancreatitis, and other unmeasured variables or unknown factors. Residual confounding by smoking could also have been present, despite the fact that accounting for smoking duration and intensity did not substantially affect the results. Misclassified diabetes status could have biased our results, but consideration of either self‐reported or validated data on type 2 diabetes status at baseline made no substantial difference in the results.

In conclusion, this study does not support an association between dietary intakes of total flavonoids, flavonoids subclasses, or lignans and pancreatic cancer risk in the EPIC cohort.

## Supporting information

Supporting Information

Supporting Information

Supporting Information

Supporting Information
