# Supplementary material for: Flavonoid and lignan intake and pancreatic cancer risk in the European prospective investigation into cancer and nutrition cohort
Source: Int J Cancer. 2016 Jun 10;139(7):1480–92. doi: 10.1002/ijc.30190 (PMC4949532; doi:10.1002/ijc.30190)
Supplement: Supplementary file 1 — Supporting Information [file IJC-139-1480-s001.doc]

| **Supplemental Table 1:** Hazard ratios (95% confidence intervals) of pancreatic cancer by quintiles of dietary intakes of flavonoids and lignans, stratified by smoking status. | | | | | | |
| --- | --- | --- | --- | --- | --- | --- |
|  | **Never Smokers (n=336)** | | **Former Smokers (n=239)** | | **Current Smokers (n=275)** | |
|  | Cases | HR (95% CI) | Cases | HR (95% CI) | Cases | HR (95% CI) |
| **Total Flavonoids** |  |  |  |  |  |  |
| Q1 | 54 | 1.00 | 45 | 1.00 | 82 | 1.00 |
| Q2 | 61 | 0.89 (0.61-1.29) | 39 | 0.78 (0.50-1.22) | 70 | 1.17 (0.84-1.63) |
| Q3 | 64 | 0.90 (0.61-1.33) | 44 | 0.95 (0.61-1.49) | 47 | 0.98 (0.66-1.45) |
| Q4 | 77 | 1.04 (0.70-1.55) | 53 | 1.05 (0.67-1.66) | 37 | 0.93 (0.60-1.44) |
| Q5 | 80 | 0.99 (0.65-1.51) | 58 | 0.99 (0.61-1.61) | 39 | 1.16 (0.73-1.84) |
| p trend |  | 0.721 |  | 0.659 |  | 0.764 |
| Continuous (log2) |  | 0.99 (0.87-1.13) |  | 1.07 (0.92-1.25) |  | 1.00 (0.88-1.14) |
| ‡ Continuous (log2) |  |  |  | 1.11 (0.95-1.29) |  | 1.05 (0.92-1.21) |
|  |  |  | p for interaction: 0.142 | |  |  |
| **Flavanols** |  |  |  |  |  |  |
| Q1 | 48 | 1.00 | 47 | 1.00 | 75 | 1.00 |
| Q2 | 64 | 1.07 (0.73-1.57) | 38 | 0.72 (0.46-1.12) | 70 | 1.27 (0.91-1.78) |
| Q3 | 67 | 1.08 (0.73-1.60) | 40 | 0.76 (0.48-1.19) | 51 | 1.14 (0.77-1.67) |
| Q4 | 76 | 1.16 (0.77-1.73) | 57 | 1.05 (0.67-1.63) | 40 | 1.10 (0.72-1.69) |
| Q5 | 81 | 1.13 (0.73-1.74) | 57 | 0.85 (0.52-1.37) | 39 | 1.27 (0.80-2.02) |
| p trend |  | 0.674 |  | 0.979 |  | 0.512 |
| Continuous (log2) |  | 0.96 (0.86-1.07) |  | 1.06 (0.93-1.21) |  | 1.01 (0.90-1.12) |
| ‡ Continuous (log2) |  |  |  | 1.09 (0.95-1.25) |  | 1.05 (0.93-1.18) |
|  |  |  | p for interaction: 0.152 | |  |  |
| **Flavan-3-ols** |  |  |  |  |  |  |
| Q1 | 53 | 1.00 | 37 | 1.00 | 61 | 1.00 |
| Q2 | 56 | 0.96 (0.64-1.43) | 45 | 0.92 (0.58-1.45) | 71 | 1.22 (0.85-1.75) |
| Q3 | 66 | 1.16 (0.77-1.76) | 48 | 0.73 (0.45-1.19) | 53 | 0.85 (0.56-1.29) |
| Q4 | 76 | 1.18 (0.77-1.81) | 49 | 0.67 (0.40-1.14) | 47 | 1.04 (0.66-1.64) |
| Q5 | 85 | 1.15 (0.72-1.83) | 70 | 1.02 (0.61-1.72) | 43 | 1.35 (0.83-2.20) |
| p trend |  | 0.670 |  | 0.293 |  | 0.167 |
| Continuous (log2) |  | 1.01 (0.94-1.08) |  | 1.03 (0.95-1.13) |  | 1.01 (0.93-1.09) |
| ‡ Continuous (log2) |  |  |  | 1.06 (0.97-1.16) |  | 1.04 (0.95-1.15) |
|  |  |  | p for interaction: 0.218 | |  |  |
| **Proanthocyanidins** |  |  |  |  |  |  |
| Q1 | 59 | 1.00 | 48 | 1.00 | 94 | 1.00 |
| Q2 | 61 | 0.82 (0.57-1.18) | 55 | 1.03 (0.69-1.53) | 62 | 0.95 (0.68-1.32) |
| Q3 | 59 | 0.78 (0.53-1.14) | 46 | 0.81 (0.53-1.24) | 48 | 0.91 (0.63-1.31) |
| Q4 | 85 | 1.17 (0.80-1.70) | 33 | 0.65 (0.40-1.04) | 28 | 0.65 (0.41-1.03) |
| Q5 | 72 | 1.04 (0.69-1.59) | 57 | 1.10 (0.69-1.75) | 43 | 0.91 (0.58-1.44) |
| p trend |  | 0.287 |  | 0.864 |  | 0.424 |
| Continuous (log2) |  | 0.93 (0.82-1.05) |  | 1.01 (0.86-1.19) |  | 0.97 (0.86-1.09) |
| ‡ Continuous (log2) |  |  |  | 1.01 (0.86-1.20) |  | 0.99 (0.87-1.13) |
|  |  |  | p for interaction: 0.546 | |  |  |
| **Theaflavins** |  |  |  |  |  |  |
| Q1 | 108 | 1.00 | 81 | 1.00 | 133 | 1.00 |
| Q2 | 67 | 1.32 (0.89-1.97) | 49 | 1.03 (0.66-1.61) | 58 | 1.02 (0.69-1.52) |
| Q3 | 76 | 1.33 (0.90-1.98) | 39 | 0.92 (0.57-1.49) | 41 | 1.00 (0.64-1.56) |
| Q4 | 85 | 1.30 (0.83-2.02) | 70 | 1.30 (0.80-2.12) | 43 | 1.34 (0.84-2.15) |
| p trend |  | 0.673 |  | 0.195 |  | 0.184 |
| Continuous (log2) |  | 1.01 (0.99-1.03) |  | 1.01 (0.99-1.03) |  | 1.00 (0.99-1.03) |
| ‡ Continuous (log2) |  |  |  | 1.02 (0.99-1.04) |  | 1.01 (0.99-1.03) |
|  |  |  | p for interaction: 0.260 | |  |  |
| **Anthocyanidins** |  |  |  |  |  |  |
| Q1 | 65 | 1.00 | 55 | 1.00 | 93 | 1.00 |
| Q2 | 77 | 1.06 (0.75-1.49) | 55 | 1.05 (0.71-1.54) | 50 | 0.82 (0.57-1.17) |
| Q3 | 68 | 0.94 (0.65-1.35) | 43 | 0.91 (0.60-1.39) | 67 | 1.32 (0.94-1.86) |
| Q4 | 61 | 0.88 (0.60-1.31) | 49 | 1.26 (0.82-1.95) | 36 | 0.87 (0.57-1.32) |
| Q5 | 65 | 1.02 (0.66-1.58) | 37 | 1.21 (0.73-2.02) | 29 | 0.81 (0.49-1.34) |
| p trend |  | 0.941 |  | 0.335 |  | 0.510 |
| Continuous (log2) |  | 0.92 (0.82-1.04) |  | 1.01 (0.87-1.16) |  | 1.01 (0.90-1.13) |
| ‡ Continuous (log2) |  |  |  | 1.00 (0.86-1.15) |  | 1.03 (0.91-1.16) |
|  |  |  | p for interaction: 0.817 | |  |  |
| **Flavonols** |  |  |  |  |  |  |
| Q1 | 61 | 1.00 | 37 | 1.00 | 67 | 1.00 |
| Q2 | 60 | 0.94 (0.65-1.36) | 50 | 1.16 (0.74-1.80) | 69 | 1.23 (0.86-1.76) |
| Q3 | 75 | 1.18 (0.81-1.71) | 45 | 0.97 (0.60-1.57) | 50 | 0.99 (0.65-1.50) |
| Q4 | 60 | 0.91 (0.60-1.38) | 46 | 1.00 (0.60-1.66) | 49 | 1.26 (0.81-1.97) |
| Q5 | 80 | 1.16 (0.75-1.79) | 61 | 1.25 (0.74-2.11) | 40 | 1.28 (0.77-2.12) |
| p trend |  | 0.488 |  | 0.390 |  | 0.387 |
| Continuous (log2) |  | 0.91 (0.78-1.06) |  | 1.08 (0.89-1.31) |  | 1.06 (0.88-1.26) |
| ‡ Continuous (log2) |  |  |  | 1.12 (0.91-1.36) |  | 1.10 (0.91-1.33) |
|  |  |  | p for interaction: 0.191 | |  |  |
| **Flavanones** |  |  |  |  |  |  |
| Q1 | 66 | 1.00 | 47 | 1.00 | 76 | 1.00 |
| Q2 | 68 | 1.03 (0.73-1.47) | 38 | 0.89 (0.57-1.38) | 59 | 0.91 (0.64-1.30) |
| Q3 | 67 | 0.93 (0.65-1.33) | 51 | 1.26 (0.83-1.91) | 47 | 0.86 (0.58-1.25) |
| Q4 | 70 | 0.89 (0.62-1.27) | 63 | 1.49 (1.00-2.22) | 51 | 0.91 (0.62-1.33) |
| Q5 | 65 | 0.85 (0.57-1.25) | 40 | 1.01 (0.63-1.62) | 42 | 0.70 (0.45-1.08) |
| p trend |  | 0.283 |  | 0.531 |  | 0.135 |
| Continuous (log2) |  | 0.97 (0.90-1.04) |  | 1.01 (0.93-1.11) |  | 0.96 (0.89-1.02) |
| ‡ Continuous (log2) |  |  |  | 1.02 (0.93-1.11) |  | 0.97 (0.90-1.04) |
|  |  |  | p for interaction: 0.445 | |  |  |
| **Flavones** |  |  |  |  |  |  |
| Q1 | 55 | 1.00 | 50 | 1.00 | 79 | 1.00 |
| Q2 | 76 | 1.23 (0.83-1.82) | 48 | 0.81 (0.53-1.23) | 54 | 1.00 (0.69-1.44) |
| Q3 | 74 | 1.11 (0.73-1.69) | 49 | 0.83 (0.53-1.28) | 48 | 1.13 (0.76-1.69) |
| Q4 | 72 | 1.13 (0.73-1.75) | 47 | 0.90 (0.58-1.42) | 49 | 1.25 (0.83-1.88) |
| Q5 | 59 | 0.95 (0.59-1.52) | 45 | 0.74 (0.45-1.21) | 45 | 0.99 (0.63-1.55) |
| p trend |  | 0.406 |  | 0.388 |  | 0.936 |
| Continuous (log2) |  | 0.95 (0.85-1.05) |  | 0.93 (0.83-1.04) |  | 1.08 (0.98-1.18) |
| ‡ Continuous (log2) |  |  |  | 0.94 (0.84-1.05) |  | 1.11 (1.00-1.23) |
|  |  |  | p for interaction: 0.201 | |  |  |
| **Isoflavones** |  |  |  |  |  |  |
| Q1 | 57 | 1.00 | 42 | 1.00 | 43 | 1.00 |
| Q2 | 62 | 0.90 (0.60-1.36) | 39 | 0.52 (0.32-0.84) | 81 | 1.08 (0.70-1.65) |
| Q3 | 70 | 1.01 (0.65-1.58) | 56 | 0.82 (0.51-1.33) | 82 | 0.96 (0.61-1.50) |
| Q4 | 78 | 1.01 (0.62-1.64) | 56 | 0.94 (0.55-1.62) | 43 | 0.63 (0.37-1.09) |
| Q5 | 69 | 1.12 (0.65-1.93) | 46 | 1.07 (0.57-2.02) | 26 | 0.81 (0.42-1.56) |
| p trend |  | 0.488 |  | 0.197 |  | 0.442 |
| Continuous (log2) |  | 1.03 (0.92-1.16) |  | 1.03 (0.89-1.20) |  | 0.89 (0.75-1.05) |
| ‡ Continuous (log2) |  |  |  | 1.04 (0.89-1.21) |  | 0.83 (0.70-1.00) |
|  |  |  | p for interaction: 0.173 | |  |  |
| **Lignans** |  |  |  |  |  |  |
| Q1 | 70 | 1.00 | 31 | 1.00 | 65 | 1.00 |
| Q2 | 58 | 0.85 (0.59-1.22) | 36 | 1.01 (0.61-1.68) | 46 | 0.80 (0.54-1.19) |
| Q3 | 72 | 1.01 (0.70-1.47) | 44 | 1.19 (0.72-2.00) | 57 | 1.09 (0.73-1.64) |
| Q4 | 63 | 0.83 (0.55-1.27) | 52 | 1.19 (0.69-2.07) | 50 | 0.85 (0.52-1.38) |
| Q5 | 73 | 0.87 (0.55-1.40) | 76 | 1.31 (0.72-2.40) | 57 | 0.89 (0.51-1.55) |
| p trend |  | 0.652 |  | 0.351 |  | 0.772 |
| Continuous (log2) |  | 0.95 (0.76-1.18) |  | 1.16 (0.89-1.53) |  | 1.02 (0.80-1.31) |
| ‡ Continuous (log2) |  |  |  | 1.16 (0.88-1.53) |  | 1.06 (0.82-1.39) |
|  |  |  | p for interaction: 0.145 | |  |  |
| Multivariable HR - adjusted for total energy intake from fat and from non-fat sources (continuous), body mass index (continuous), alcohol intake (non drinkers, drinkers of 0-6 g/d, >6-12 g/d, >12-24g/d, >24-60 g/d, women drinkers of: >60g/d, men drinkers of: >60-96 g/d, >96 g/d), diabetes status at recruitment (yes, diagnosis verified; yes, diagnosis self-reported; not diabetic; missing status), and stratified by age (1-year categories), sex, and centre. | | | | | | |
| ‡ Additionally controlling for time since quitting smoking (in former smokers, with 228 pancreatic cancer cases available for analysis), or duration and number of cigarettes/day (in current smokers, with 239 pancreatic cancer cases available for analysis). | | | | | | |
