# Supplementary material for: Flavonoid and lignan intake and pancreatic cancer risk in the European prospective investigation into cancer and nutrition cohort
Source: Int J Cancer. 2016 Jun 10;139(7):1480–92. doi: 10.1002/ijc.30190 (PMC4949532; doi:10.1002/ijc.30190)
Supplement: Supplementary file 2 — Supporting Information [file IJC-139-1480-s002.doc]

| **Supplemental Table 2**: Sensitivity analyses on the association between dietary intakes of flavonoids subclasses and pancreatic cancer risk. | | | | | | | |
| --- | --- | --- | --- | --- | --- | --- | --- |
|  | Quintile of intake | | | | | | Intake as continuous variable (log2) |
|  | Q1 | Q2 | Q3 | Q4 | Q5 | *p for trend* |  |
| **Flavanols** |  |  |  |  |  |  |  |
| *Non-Diabetics* |  |  |  |  |  |  |  |
| Cases | 156 | 151 | 141 | 160 | 165 |  |  |
| HR (95% CI) | 1.00 | 0.98 (0.78-1.23) | 0.95 (0.74-1.21) | 1.07 (0.83-1.37) | 1.03 (0.78-1.34) | 0.677 | 0.98 (0.92-1.05) |
| *Microscopically confirmed* |  |  |  |  |  |  |  |
| Cases | 143 | 136 | 123 | 108 | 98 |  |  |
| HR (95% CI) | 1.00 | 1.07 (0.84-1.36) | 1.11 (0.86-1.44) | 1.08 (0.82-1.43) | 1.22 (0.90-1.65) | 0.245 | 1.00 (0.93-1.08) |
| *> 2 years follow-up* |  |  |  |  |  |  |  |
| Cases | 148 | 158 | 145 | 158 | 168 |  |  |
| HR (95% CI) | 1.00 | 1.10 (0.88-1.39) | 1.08 (0.85-1.39) | 1.19 (0.92-1.53) | 1.17 (0.89-1.54) | 0.345 | 1.01 (0.95-1.09) |
|  |  |  |  |  |  |  |  |
| **Flavan-3-ols** |  |  |  |  |  |  |  |
| *Non-Diabetics* |  |  |  |  |  |  |  |
| *Cases* | 132 | 152 | 152 | 149 | 188 |  |  |
| HR (95% CI) | 1.00 | 1.03 (0.80-1.31) | 0.94 (0.72-1.22) | 0.98 (0.74-1.29) | 1.20 (0.90-1.61) | 0.082 | 1.02 (0.97-1.06) |
| *Microscopically confirmed* |  |  |  |  |  |  |  |
| Cases | 121 | 134 | 137 | 112 | 104 |  |  |
| HR (95% CI) | 1.00 | 0.98 (0.76-1.27) | 0.95 (0.72-1.26) | 0.92 (0.68-1.24) | 1.17 (0.84-1.61) | 0.183 | 1.01 (0.96-1.07) |
| *> 2 years follow-up* |  |  |  |  |  |  |  |
| Cases | 135 | 155 | 155 | 142 | 190 |  |  |
| HR (95% CI) | 1.00 | 1.03 (0.81-1.32) | 0.97 (0.75-1.26) | 0.97 (0.73-1.27) | 1.23 (0.92-1.65) | 0.071 | 1.02 (0.98-1.07) |
|  |  |  |  |  |  |  |  |
| **Proanthocyanidins** |  |  |  |  |  |  |  |
| *Non-Diabetics* |  |  |  |  |  |  |  |
| Cases | 187 | 160 | 142 | 130 | 154 |  |  |
| HR (95% CI) | 1.00 | 0.88 (0.71-1.10) | 0.80 (0.64-1.01) | 0.80 (0.62-1.02) | 0.94 (0.72-1.23) | 0.790 | 0.93 (0.86-1.01) |
| *Microscopically confirmed* |  |  |  |  |  |  |  |
| Cases | 165 | 135 | 104 | 96 | 108 |  |  |
| HR (95% CI) | 1.00 | 0.98 (0.77-1.23) | 0.86 (0.67-1.12) | 0.98 (0.74-1.29) | 1.10 (0.82-1.48) | 0.498 | 0.97 (0.89-1.06) |
| *> 2 years follow-up* |  |  |  |  |  |  |  |
| Cases | 180 | 162 | 148 | 130 | 157 |  |  |
| HR (95% CI) | 1.00 | 0.94 (0.76-1.17) | 0.89 (0.71-1.12) | 0.87 (0.67-1.11) | 1.05 (0.81-1.37) | 0.646 | 0.97 (0.89-1.06) |
|  |  |  |  |  |  |  |  |
| **Theaflavins** |  |  |  |  |  |  |  |
| *Non-Diabetics* |  |  |  |  |  |  |  |
| Cases | 279 | 161 | 145 | 188 |  |  |  |
| HR (95% CI) | 1.00 | 1.12 (0.88-1.44) | 1.09 (0.84-1.41) | 1.32 (1.00-1.74) |  | 0.067 | 1.01 (0.99-1.02) |
| *Microscopically confirmed* |  |  |  |  |  |  |  |
| Cases | 265 | 132 | 108 | 103 |  |  |  |
| HR (95% CI) | 1.00 | 1.03 (0.79-1.36) | 0.98 (0.74-1.32) | 1.21 (0.89-1.66) |  | 0.200 | 1.00 (0.99-1.02) |
| *> 2 years follow-up* |  |  |  |  |  |  |  |
| Cases | 295 | 153 | 139 | 190 |  |  |  |
| HR (95% CI) | 1.00 | 1.07 (0.83-1.37) | 1.04 (0.80-1.35) | 1.30 (0.98-1.71) |  | 0.055 | 1.01 (0.99-1.02) |
|  |  |  |  |  |  |  |  |
| **Anthocyanidins** |  |  |  |  |  |  |  |
| *Non-Diabetics* |  |  |  |  |  |  |  |
| Cases | 193 | 169 | 165 | 131 | 115 |  |  |
| HR (95% CI) | 1.00 | 0.99 (0.80-1.23) | 1.06 (0.85-1.32) | 0.95 (0.74-1.22) | 0.92 (0.69-1.23) | 0.522 | 0.96 (0.90-1.04) |
| *Microscopically confirmed* |  |  |  |  |  |  |  |
| Cases | 179 | 130 | 123 | 98 | 78 |  |  |
| HR (95% CI) | 1.00 | 0.94 (0.75-1.19) | 1.07 (0.84-1.37) | 1.06 (0.81-1.40) | 1.01 (0.73-1.39) | 0.777 | 0.99 (0.91-1.07) |
| *> 2 years follow-up* |  |  |  |  |  |  |  |
| Cases | 185 | 170 | 170 | 137 | 115 |  |  |
| HR (95% CI) | 1.00 | 1.06 (0.86-1.32) | 1.18 (0.94-1.47) | 1.09 (0.85-1.40) | 1.04 (0.78-1.39) | 0.899 | 1.00 (0.92-1.07) |
|  |  |  |  |  |  |  |  |
| **Flavonols** |  |  |  |  |  |  |  |
| *Non-Diabetics* |  |  |  |  |  |  |  |
| Cases | 148 | 163 | 149 | 144 | 169 |  |  |
| HR (95% CI) | 1.00 | 1.12 (0.89-1.41) | 1.06 (0.82-1.36) | 1.08 (0.82-1.41) | 1.25 (0.94-1.66) | 0.157 | 1.00 (0.90-1.11) |
| *Microscopically confirmed* |  |  |  |  |  |  |  |
| Cases | 135 | 140 | 134 | 98 | 101 |  |  |
| HR (95% CI) | 1.00 | 1.13 (0.88-1.44) | 1.19 (0.91-1.56) | 1.07 (0.79-1.46) | 1.32 (0.96-1.83) | 0.168 | 0.99 (0.88-1.11) |
| *> 2 years follow-up* |  |  |  |  |  |  |  |
| Cases | 144 | 161 | 160 | 138 | 174 |  |  |
| HR (95% CI) | 1.00 | 1.13 (0.90-1.43) | 1.17 (0.92-1.51) | 1.08 (0.82-1.42) | 1.35 (1.01-1.79) | 0.072 | 1.02 (0.92-1.13) |
|  |  |  |  |  |  |  |  |
| **Flavanones** |  |  |  |  |  |  |  |
| *Non-Diabetics* |  |  |  |  |  |  |  |
| Cases | 172 | 155 | 151 | 164 | 131 |  |  |
| HR (95% CI) | 1.00 | 0.99 (0.79-1.23) | 1.00 (0.79-1.26) | 1.01 (0.81-1.27) | 0.82 (0.63-1.06) | 0.132 | 0.97 (0.93-1.02) |
| *Microscopically confirmed* |  |  |  |  |  |  |  |
| Cases | 145 | 132 | 103 | 132 | 96 |  |  |
| HR (95% CI) | 1.00 | 0.94 (0.74-1.20) | 0.85 (0.65-1.11) | 0.98 (0.76-1.26) | 0.71 (0.53-0.95) | 0.045 | 0.96 (0.91-1.00) |
| *> 2 years follow-up* |  |  |  |  |  |  |  |
| Cases | 172 | 155 | 152 | 167 | 131 |  |  |
| HR (95% CI) | 1.00 | 1.01 (0.80-1.26) | 1.03 (0.82-1.29) | 1.06 (0.85-1.33) | 0.81 (0.63-1.05) | 0.124 | 0.97 (0.93-1.01) |
|  |  |  |  |  |  |  |  |
| **Flavones** |  |  |  |  |  |  |  |
| *Non-Diabetics* |  |  |  |  |  |  |  |
| Cases | 170 | 161 | 163 | 148 | 131 |  |  |
| HR (95% CI) | 1.00 | 1.01 (0.80-1.27) | 1.05 (0.82-1.34) | 1.04 (0.80-1.35) | 0.88 (0.66-1.17) | 0.313 | 0.98 (0.92-1.05) |
| *Microscopically confirmed* |  |  |  |  |  |  |  |
| Cases | 166 | 127 | 105 | 106 | 104 |  |  |
| HR (95% CI) | 1.00 | 1.01 (0.79-1.29) | 1.03 (0.78-1.36) | 1.06 (0.80-1.40) | 0.89 (0.65-1.20) | 0.394 | 1.00 (0.93-1.06) |
| *> 2 years follow-up* |  |  |  |  |  |  |  |
| Cases | 170 | 166 | 162 | 153 | 126 |  |  |
| HR (95% CI) | 1.00 | 1.04 (0.83-1.31) | 1.04 (0.82-1.33) | 1.07 (0.83-1.38) | 0.83 (0.62-1.10) | 0.121 | 0.98 (0.92-1.04) |
|  |  |  |  |  |  |  |  |
| **Isoflavones** |  |  |  |  |  |  |  |
| *Non-Diabetics* |  |  |  |  |  |  |  |
| Cases | 116 | 168 | 189 | 165 | 135 |  |  |
| HR (95% CI) | 1.00 | 0.89 (0.69-1.17) | 0.97 (0.74-1.29) | 0.92 (0.67-1.26) | 1.05 (0.73-1.51) | 0.399 | 1.00 (0.92-1.09) |
| *Microscopically confirmed* |  |  |  |  |  |  |  |
| Cases | 103 | 148 | 175 | 120 | 62 |  |  |
| HR (95% CI) | 1.00 | 0.86 (0.65-1.14) | 1.01 (0.75-1.35) | 0.84 (0.60-1.18) | 0.98 (0.65-1.48) | 0.795 | 1.01 (0.91-1.12) |
| *> 2 years follow-up* |  |  |  |  |  |  |  |
| Cases | 123 | 170 | 189 | 161 | 134 |  |  |
| HR (95% CI) | 1.00 | 0.89 (0.68-1.15) | 0.93 (0.71-1.23) | 0.85 (0.62-1.16) | 0.97 (0.68-1.40) | 0.578 | 0.98 (0.90-1.07) |
|  |  |  |  |  |  |  |  |
| Multivariable HR - adjusted for total energy intake from fat and from non-fat sources (continuous), body mass index in kg/m^2^ (continuous), smoking status and intensity (never; current, 1-15 cigarettes per day, 16-25 cigarettes per day, 26+ cigarettes per day; former, quit ≤10 years, quit 11-20 years, quit 20+ years; current, pipe/cigar/occasional; current/former, missing; unknown), alcohol intake (non drinkers; drinkers of 0-6 g/d, >6-12 g/d, >12-24g/d, >24-60 g/d; women drinkers of: >60g/d; men drinkers of: >60-96 g/d, >96 g/d), diabetes status at recruitment (yes, diagnosis verified; yes, diagnosis self-reported; not diabetic, missing status), and stratified by age (1-year categories), sex, and centre. | | | | | | | |
| *Theaflavins: Group 1: 193,832; Group 2: 93,561; Group 3: 94,386; Group 4: 95,423. | | | | | | | |

PY: person-years
