# Supplementary material for: Flavonoid and lignan intake and pancreatic cancer risk in the European prospective investigation into cancer and nutrition cohort
Source: Int J Cancer. 2016 Jun 10;139(7):1480–92. doi: 10.1002/ijc.30190 (PMC4949532; doi:10.1002/ijc.30190)
Supplement: Supplementary file 3 — Supporting Information [file IJC-139-1480-s003.doc]

**Supplemental Table 3**: Summary of all published studies on intake of dietary flavonoids and pancreatic cancer risk.

| **Study [reference]**  **Country**  **Study design** | **Study population** | **N pancreatic cancer cases**  **(follow-up in years)** | **Dietary assessment method**  **Subclasses of flavonoids studied**  **Estimation of flavonoids intake** | **Intake comparison,**  **extreme quintiles** | **Results** | |
| --- | --- | --- | --- | --- | --- | --- |
|  |  |  |  |  | HR/OR comparing extreme categories (95% CI) | Confounding variables |
| **Current study**  **Europe**  **PROSPECTIVE** | 477,309 men and women, aged > 20 years.  EPIC | 865  (11.3) | Various types of dietary questionnaires.  Total flavonoids and 9 subclasses (flavanols, flavan-3-ols, proanthocyanidins, theaflavins anthocyanidins flavanones, flavonols, flavones and isoflavones), estimated from the USDA database and from the Phenol Explorer database.  Intake was calculated using data on food content, also considering standard recipes and retention factors for processed food. | mg/day (medians)  Total flavonoids: 124.2 to 933.9  Flavanols: 83.5 to 823.5  Flavan-3-ols: 13.0 to 525.1  Proanthocyanidins: 57.6 to 312.3  Theaflavins: 0 to 19.3  Anthocyanidins: 7.8 to 56.1  Flavonols: 10.1 to 52.1  Flavanones: 3.2 to 46.3  Flavones: 0.6 to 7.3  Isoflavones: 0.2 to 2.6 | Total Flavonoids: No association.  HR = 1.10 (0.85-1.42)  Subclasses : No association.  Flavanols: 1.13 (0.87-1.46)  Flavan-3-ols: 1.23 (0.93-1.62)  Proanthocyanidins: 1.02 (0.80-1.31)  Theaflavins: 1.35 (1.03-1.75)  Anthocyanidins: 1.02 (0.78-1.33)  Flavonols: 1.31 (1.00-1.72)  Flavanones: 0.84 (0.66-1.07)  Flavones: 0.95 (0.73-1.25)  Isoflavones: 0.91 (0.64-1.28) | Age, center, sex, energy intake, BMI, smoking, alcohol use, diabetes status (validated and or self-reported). |
| Arem *et al*, 2013  [13]  USA  PROSPECTIVE | 537,104 men and women, aged 50–71 years  National Institutes of Health-AARP Diet and Health Study Cohort. | 2,379  (10.6) | FFQ  Total flavonoids and 6 subclasses (flavan-3-ols, flavanones, flavonols, anthocyanidins, flavones and isoflavones), estimated from the USDA flavonoids database.  Intake was calculated using data on food content also considering standard recipes and retention factors for processed food. | mg/day per 1000 kcal (medians)  Total flavonoids: 34.3 to 388.0  Flavan-3-ols: 8.6 to 331.2  Anthocyanidins: 2.1 to 15.2  Flavonols: 4.5 to 21.2  Flavanones: 2.6 to 55.4  Flavones: 0.2 to 1.6  Isoflavones: 0.1 to 0.5 | Total Flavonoids: No association.  HR = 1.09 (0.96–1.24)  Sublasses: No association.  Flavan-3-ols: 1.03 (0.91–1.17)  Anthocyanidins: 1.10 (0.96–1.27)  Flavonols: 1.09 (0.95–1.24)  Flavanones: 1.06 (0.92–1.21)  Flavones: 1.09 (0.95–1.25)  Isoflavones: 0.96 (0.84–1.09) | Age, sex, energy intake, smoking, BMI, alcohol use, diabetes status (self-reported), saturated fat and red meat intake. |
| Wang *et al*, 2009  [9]  USA  PROSPECTIVE | 38,408 women, aged > 45 years  Women’s health study. | Number not provided  (11.5) | FFQ  Total flavonoids and 3 flavonols (quercetin, kaempferol and myricetin) and 2 flavones (apigenin and luteolin) subclasses, estimated from published values from Europe (analyses completed in The Netherlands). | mg/day (medians)  Total flavonoids: 8.8 to 47.44  Quercetin: 6.5 to 32.8  Kaempferol: 0.9 to 13.1  Myricetin: 0.1 to 2.8  Apigenin: 0.1 to 1.4  Luteolin: 0.01 to 0.2 | Total Flavonoids: No association.  HR = not provided  Sublasses: No association.  HR = not provided | Age, race, energy intake, intervention group, smoking, alcohol use, physical activity, menopausal status, hormone replacement therapy, multivitamin use, BMI, family history of cancer, intake of fruits & vegetables, fiber, folate and saturated fat. |
| Cutler *et al*, 2008  [15]  USA  PROSPECTIVE | 34,708 postmenopausal women, aged > 55 years  Iowa Women’s Health Study. | 230  (8) | FFQ  Total flavonoids and 7 subclasses (flavan-3-ols, flavanones, flavonols, anthocyanidins, flavones, isoflavones and proanthocyanidings), estimated from the USDA flavonoids database.  Intake calculated using data on food content and also considering standard recipes. | mg/day (means)  Total flavonoids: 91 to 680  Flavan-3-ols: 4.1 to 314.6  Proanthocyanidins: 58.2 to 591.0  Flavonols: 3.9 to 23.2  Flavanones: 7.4 to 107.2  Anthocyanidins: 45.6 to 365.1  Flavones: 0.1 to 1.75  Isoflavones: 0.1 to 1.8 | Total Flavonoids: No association.  HR = not provided  Subclasses: No association.  HR = not provided | Age, race, energy intake, education,  BMI, multivitamin use, physical activity, smoking. |
| Bobe *et al*, 2008  [14]  Finland  PROSPECTIVE | 27,111 healthy male smokers, aged 50-69 years  The ATBC study | 306  (16.1) | Diet history questionnaire  Total flavonoids and 3 subclasses (flavonols, flavan-3-ols and flavones) and seven subgroups (kaempferol, myricetin, quercetion, catechin, epicatechin, apigenin and luteolin), estimated from published values from Europe (analyses completed in The Netherlands).  Intake calculated using data on food content. | mg/day, energy adjusted (range of intake in mg)  Total flavonoids: < 6.5 to > 22.9  Flavonols: < 5.2 to > 13.0  Kaempferol: < 0.2 to > 1.9  Myrecetin: <0.4 to > 1.3  Quercetin: < 4.4 to > 9.9  Flavan-3-ols: < 0.9 to > 9.8  Catechin: < 0.4 to > 3.7  Epicatechin: < 0.5 to > 6.2  Flavones: < 0.03 to > 0.2  Apigenin: < 0.02 to > 0.12  Luteolin: < 0.01 to > 0.07 | Total Flavonoids: No association.  HR = 0.90 (0.64–1.28)    Inverse association in smokers not consuming supplements: HR = 0.36 (0.17-0.78)  Subclasses: No association.  Flavonols: 0.91 (0.64-1.30)  Kaempferol: 0.93 (0.65-1.33)  Myrecetin: 1.04 (0.73-1.49)  Quercetin: 1.07 (0.75-1.53)  Flavan-3-ols: 0.92 (0.64-1.31)  Catechin: 0.95 (0.66-1.36)  Epicatechin: 0.90 (0.63-1.28)  Flavones: 0.99 (0.70-1.42)  Apigenin: 1.06 (0.74-1.51)  Luteolin: 1.09 (0.77-1.56) | Age, energy intake, diabetes status (self-reported), saturated fat. |
| Nöthlings *et al*, 2007  [17]  USA  PROSPECTIVE | 183,518 men and women, aged 45-75 years  Multiethnic cohort (MEC) study. | 529  (8) | FFQ  Flavonols intake and 3 subclasses (quercetin, myricetin and kaempferol), estimated from Hawaiian food composition database and published values of Hawaiian food.  Intake calculated using data on food content. | mg/day (means)  Flavonols: 14.83  Quercetin: 10.1  Myricetin: 0.9  Kaempferol: 3.9 | Total Flavonoids as Flavonols: HR = 0.77 (0.58-1.03; p trend=0.046)  Subclasses: No association.  Quercetin: 0.80 (0.60-1.06)  Myricetin: 0.78 (0.58-1.05)  Kaempferol: 0.85 (0.65-1.12)  Inverse associations in smokers: Flavonols: 0.41 (0.22-0.74)  Quercetin: 0.55 (0.30-0.99)  Myricetin: 0.27 (0.14-0.55)  Kaempferol: 0.55 (0.30-1.01) | Age, sex, energy intake, BMI, smoking, family history of pancreatic cancer, diabetes status (self-reported), red and processed meat. |
| Arts *et al*, 2002  [12]  USA  PROSPECTIVE | 34,651 postmenopausal women, aged > 55 years  Iowa Women´s Health Study. | 130  (13) | FFQ  Catechins and 6 subclasses (catechin, gallocatechin, epicatechin, epigallocatechin, epicatechin gallate, and epigallocatechin gallate), estimated from published values from Europe (analyses completed in The Netherlands).  Intake calculated using data on food content and also considering standard recipes. | mg/day, adjusted for energy intake (means)  Total Catechins: 3.6 to 75.1  Catechin: 1.4 to 6.1  Epicatechin: 1.9 to 18.2  Gallocatechin: 0 to 3.5  Epigallocatechin: 0 to 24.0  Epicatechin gallate: 0.2 to 17.8  Epigallocatechin gallate: 0 to 24.3  Flavonols + flavones: 5.3 to 31.4 | Total Flavonoids (catechins): No association.  HR = 0.74 (0.46–1.20)  Sublasses: No association.  HR = not provided | Age, energy intake, BMI, waist to hip ratio, physical activity, smoking, alcohol use, fruits and vegetables. |
| Knekt *et al*, 1997  [16]  Finland  PROSPECTIVE | 9,959 Finnish men and women, aged > 15 years  The Finnish Mobile Clinic Health Examination survey (1967-1991). | 29  (24) | Diet history questionnaire  Total flavonoids and 5 subclasses (myricetin, kaempferol, quercetin, luteolin and apigenin), estimated from published values from Europe (analyses completed in The Netherlands).  Intake calculated using data on food content. | mg/day (means)  Total flavonoids: ranging from 0 to 41.4  Mean intake: 24.2  Quercetin: 3.3  Kaempferol: 0.6  Myricetin: 0.12  Naringenin: 5.1  Hesperetin: 15.1 | Total Flavonoids: No association.  HR = 1.46 (0.37-5.71)  Sublasses: No association.  HR = not provided | Age, center/area, sex, occupation, BMI, smoking, energy intake, vitamin C, vitamin E, carotene, fiber, saturated, monounsaturated and polyunsaturated fatty acids, and cholesterol. |
| Rossi *et al*, 2012  [18]  Italy  CASE-CONTROL | 326 incident pancreatic cancer cases and 652 matched controls, aged 34–80 years | 326  (NA) | FFQ  Total flavonoids and 7 flavonoid subclasses (flavanols, flavanones, flavonols, anthocyanidins, flavones, and proanthocyanidins, also including 6 classes: monomers, dimers, trimers, 4–6 mers, 7–10 mers, >10 mers), estimated from the USDA flavonoids database and other published values (vegetables).  Intake calculated using data on food content. | mg/day, adjusted for energy intake (means), among controls  The mean daily intake among controls was Flavanols: 63.4  Flavonones: 36.2  Flavonols: 24.7  Anthocyanidins: 18.6  Flavones: 0.5  Pronathocyanidines: 103.5 for monomer and dimer proanthocyanidins combined, and 261.7 three or more mers | Total Flavonoids: not provided.  Subclasses : No association, except for proanthocyanidins: OR = 0.41 (0.24-0.69)  Flavanols: 0.63 (0.38–1.03)  Flavanones: 0.68 (0.41–1.14)  Flavonols: 0.69 (0.42–1.13)  Anthocyanidins 0.83 (0.43–1.60)  Flavones: 0.88 (0.53–1.46)  Proanthocyanidins: 0.41 (0.24–0.69) | Age, center, sex, non-alcohol energy intake, smoking, alcohol use, year of interview, education and diabetes status (self-reported). |

NA: Not Applicable

FFQ= Food Frequency Questionnaire

HR: Hazard ratio; OR: Odds ratio

BMI: body mass index

USDA: U.S. Department of Agriculture
