# Supplementary material for: Flavonoid and lignan intake and pancreatic cancer risk in the European prospective investigation into cancer and nutrition cohort
Source: Int J Cancer. 2016 Jun 10;139(7):1480–92. doi: 10.1002/ijc.30190 (PMC4949532; doi:10.1002/ijc.30190)
Supplement: Supplementary file 4 — Supporting Information [file IJC-139-1480-s004.docx]

**Supplemental Tables**:

**Supplemental Table 1**: Hazard ratios (95% confidence intervals) of pancreatic cancer by quintiles of dietary intakes of flavonoids and lignans, stratified by smoking status.

**Supplemental Table 2**: Sensitivity analyses on the association between dietary intakes of flavonoids subclasses and pancreatic cancer risk.

**Suppmentary Table 3**: Summary of all pubslihed studies on intake of dietary flavonoids and pancreatic cancer risk.
